# A tough 3D puzzle in the walnut shell

**DOI:** 10.1093/jxb/erab221

**Published:** 2021-06-22

**Authors:** Rivka Elbaum, Michael Elbaum

**Affiliations:** 1 The Smith Institute of Plant Sciences and Genetics in Agriculture, The Hebrew University of Jerusalem, Rehovot, Israel; 2 Department of Chemical and Biological Physics, Weizmann Institute of Science, Rehovot, Israel

**Keywords:** Cell growth, 3D imaging, puzzle cells, seed shell, serial surface microscopy

## Abstract

This article comments on:

**Antreich SJ, Xiao N, Huss JC, Gierlinger N.** 2021. A belt for the cell: cellulosic wall thickenings and their role in morphogenesis of the 3D puzzle cells in walnut shells. Journal of Experimental Botany **72,**4744–4756.

**Plant organs initiate as a group of tiny meristematic cells. After expansion, three basic shapes of organs may be defined: cylindrical, laminar, and spherical (** Trinh *et al.*, 2021 **). The development of a lamina can be followed by various surface and subsurface microscopy methods. However, organs shaped as opaque spheres are most conveniently studied in sections.** Antreich *et al.* (2021 **) applied 3D reconstructions based on optical and scanning electron microscopy to study the development of cells building the walnut shell. Restricted by cellulose, the growing cells bulge and interdigitate with neighboring cells, leaving gaps at the regions of high curvature. Examining the cell interfaces with Raman microspectroscopy, they show that these gaps are lined with pectin.**

The imbalance between cell wall extensibility and turgor pressure is the main driving force for plant cell growth. Organs shaped as cylinders, laminae, and spheres can be created by a group of cells that expand in one, two, or three directions. The hydrostatic turgor pressure is uniform within the protoplast volume, implying that a similar stress should act in all directions within the cell. Therefore, cells may grow into complex shapes according to local variations in the cell wall stiffness: a locally soft region of cell wall would stretch into a bulging lobe under the internal pressure.

Cellulose microfibrils resist extension. The common paradigm is thus that cell walls elongate perpendicularly to the cellulose microfibril average orientation. In Arabidopsis leaves, epidermis cells are shaped as flat jigsaw-puzzle parts, forming a continuous 2D lamina. The formation of lobes has been monitored by various microscopy methods in live tissues of the wild type and cell wall mutants, as well as under drug treatments. It was shown that lobe development includes two stages: initiation and expansion. Initiation is not related to cellulose microfibril alignment. Instead, local concentrations of de-methylesterified pectin stiffen the wall. Under expansion, the pectin-rich regions create concave undulations. In order for the undulation to grow into a bulge, stiff cellulose microfibrils are aligned there, forming a fan. The cellulose structure restricts extension in the leaf plane and thus stabilizes the initiated lobe, enabling its further development (Altartouri *et al.*, 2019). A debate was raised with the suggestion that pectin may crystallize into filaments at the convex walls. These pectin nanofibrils may align perpendicularly to the leaf surface, swell in the leaf plane as they de-esterify, and add to the developing lobes by extending the cell wall at its convex tip (Cosgrove and Anderson, 2020; Haas *et al.*, 2020).

Can this model explain the formation of the 3D jigsaw-puzzle cells in the hard walnut shell, as shown by Antreich *et al.* (2021)? An obvious difference is the third dimension—these cells expand in all directions and fill a 3D space. An additional difference is the stress direction. While the 2D jigsaw-puzzle shaped cells of the leaf epidermis should resist stress in the plane, the 3D nut shell is made to withstand impact coming from outside the organ. At the same time, the shell structure must allow for disassembly by pressure of the germinating seed from within. In order to visualize the development of the opaque thick shell tissue, Antreich *et al.* (2021) applied various microscopy methods by which they could follow the growth of small cells into polylobate sclereids. The approach is that of multiscale serial surface imaging using a microtome and either a macro-camera or a scanning electron microscope (Box 1). By iteratively trimming and imaging the exposed surface, the investigators could build 3D models of the nut shell at both centimetre and micron scales. In addition to the morphological imaging of the cells and cell walls, Raman microspectroscopy (Box 2) was applied to sections at strategic locations to distinguish among the key components: cellulose, hemicellulose, and pectin.

**Box 1. Serial surface imaging**

How can we look inside a nut, a bone, or any other opaque specimen? One approach is to cut sections sufficiently thin for transparency. Their images can be ordered in sequence and aligned to create a 3D view. This is the classic approach of serial sections in TEM, updated recently as array tomography in SEM. An alternative approach is to remove sections but to instead observe iteratively the newly exposed surfaces of the remaining sample. An advantage is that the integrity of the section is not important. Different technologies for such serial surface imaging go by different names. For example, serial block face (SBF) when a microtome is mounted inside the SEM, or, when the microtome is replaced by a focused ion beam (FIB) mill, as 3D FIB-SEM. The work by Antreich *et al*. is an elegant example of multimodal 3D imaging combining the strengths of X-ray tomography (micro-CT) with serial surface approaches using both SEM and visible light macro-photography.

**Box 2. Raman microspectroscopy**

Raman spectroscopy provides a method to probe the composition of a sample, based on the vibrational movements of the constituent molecules. In a nutshell, the electron clouds that bond atoms in a molecule can contract and extend, like a mass on a spring, each with a characteristic frequency and associated energy. Illuminating photons of higher energy may excite these oscillations, in which case the scattered photons will be less energetic. The difference in energy between the incoming and scattered photons is the Raman effect. Characteristic energies identify bonds and characteristic patterns identify molecules such as cellulose, pectin, etc. Using a laser, we can focus a stream of photons to a point in our sample and measure the Raman spectrum. In Raman microspectroscopy, we collect spectra point by point to form a microscopic image of the molecular vibrations.

This work traces the ~13-fold increase in volume of the fruit over 4 weeks of development, from week 6 to 10 after flowering. During this period, the polyhedron cells expand into jigsaw-puzzle cells. Cellulose hoops that form stiff regions restrict the growth of the cell wall. As the wall expands through the hoop, a concave bend is left behind and a gap opens up between neighbouring cells. The neighbouring convex cell wall presents a high content of esterified pectin. This may loosen the contact between the cells (Fig. 1).

**Fig. 1.**
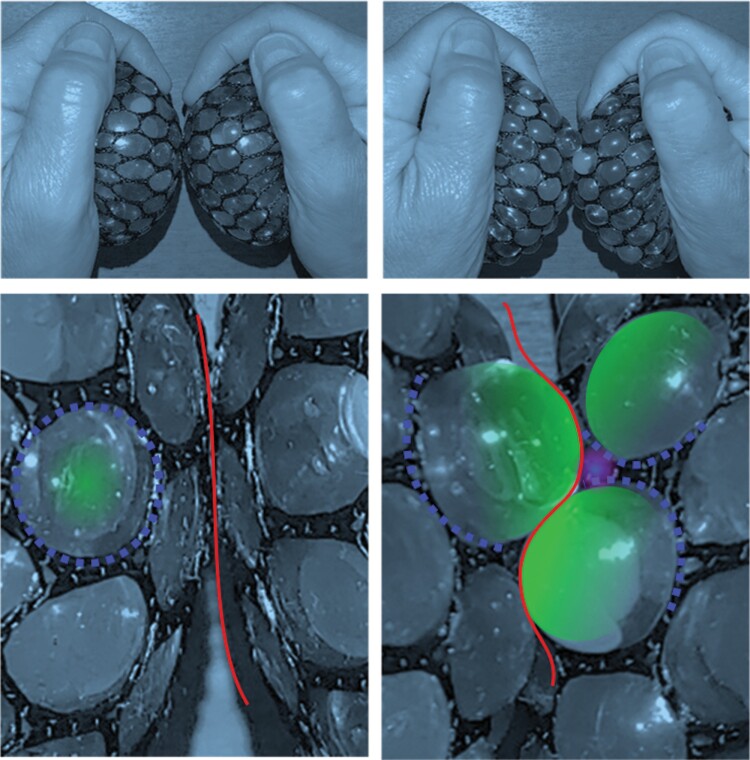
Stress-ball toy demonstrating the bulging and gaps. A flexible ball is covered by stiff net. (A) Under balanced pressure, the faces of the ball are smooth, similarly to the state in a young polyhedron cell. (B) A close-up showing the interface between the balls (red line). A blue circle represents a loop of crystalline cellulose around a softer portion of the wall with high content of pectin (green). (C) When pressure is applied on the ball, its elastic membrane bulges through the net. (D) A close-up showing the undulating interface between two balls (red line). The softer parts, which represent the pectin-rich wall (green), create the convex regions, while the stiffer parts, which represent the cellulose (blue), remain in the concave parts. At these stiff regions, gaps form between the two balls (purple haze), similarly to the gaps forming between the cells.

Once the cells reach their final size, the cell wall begins to accumulate lignin. Lignification starts in the external parts of the shell and advances inward toward the thinner walled cells close to the seed (Xiao *et al.*, 2020). The lignin monomers are accumulated at the cell’s membrane, and possibly transferred to the corners and middle lamella for impregnation into the wall. Raman microspectroscopy showed the presence of lignin or lignin monomers in the pits. The numerous pits may have a role in extending the cell membrane surface, and specifically enabling the transport of lignin monomers to the older parts of the cell wall, localized far from the plasma membrane. The authors suggest that the intercellular gaps possibly allow fast distribution of lignin monomers and lignification enzymes between cells.

Is there a mechanical function for the gaps? In the jigsaw-puzzle Arabidopsis pavement cells, there are no gaps. Neighbouring cell contact length is increased significantly by the wavy shape, possibly increasing stability under mechanical strains in the leaf plane. In contrast, the gaps between the cells in the walnut shell reduce the contact area (figs 3 and 4B in Antreich *et al.*, 2021). Apparently, the lobes that interlock the cells to one another are sufficient to maintain the strength of the shell (Antreich *et al.*, 2019). As the main role of the shell is to protect the seed from external shocks, the gaps may allow some relative movement to absorb and dissipate the impact. This structural flexibility in the 3D puzzle should increase mechanical toughness as the shell is compressed so as to avoid brittle fracture. If the forces originate from within, however, expanding the diameter of the shell rather than contracting it, the lobes may slip past one another to allow the shell to open. Combined with hydration, the interdigitating lobe structure provides an elegant mechanism for the asymmetry in resistance to forces from outside or from within, which is so necessary to the protective role of the nut shell (Huss *et al.*, 2020).

In summary, the developmental profile shown by Antreich *et al.* (2021) extends our view beyond the well-studied 1D (cylindrical hypocotyl) and 2D (laminated leaf) growing directions. The imaging approaches also extend the possibilities to study diverse plant samples, including opaque or woody specimens. Illumination of the 3D interlocked structure of the shell may inspire the construction of lightweight coverings such as shields or even roofs.
